# Resistance Training Modulates Reticulum Endoplasmic Stress, Independent of Oxidative and Inflammatory Responses, in Elderly People

**DOI:** 10.3390/antiox11112242

**Published:** 2022-11-14

**Authors:** Brisamar Estébanez, Nishant P. Visavadiya, José E. Vargas, Marta Rivera-Viloria, Andy V. Khamoui, José A. de Paz, Chun-Jung Huang

**Affiliations:** 1 Institute of Biomedicine (IBIOMED), University of León, 24007 León, Spain; 2 Exercise Biochemistry Laboratory, Department of Exercise Science and Health Promotion, Florida Atlantic University, Boca Raton, FL 33431, USA; 3 Department of Cell Biology, Federal University of Paraná, Curitiba 81531-980, Paraná, Brazil; 4 Institute for Human Health and Disease Intervention, Florida Atlantic University, Boca Raton, FL 33458, USA; 5 Stiles-Nicholson Brain Institute, Florida Atlantic University, Boca Raton, FL 33458, USA

**Keywords:** aging, exercise, HSP60, inflammaging, IRE1, Klotho, oxidative stress, strength exercise, systems biology, UPR

## Abstract

Aging is related to changes in the redox status, low-grade inflammation, and decreased endoplasmic reticulum unfolded protein response (UPR). Exercise has been shown to regulate the inflammatory response, balance redox homeostasis, and ameliorate the UPR. This work aimed to investigate the effects of resistance training on changes in the UPR, oxidative status, and inflammatory responses in peripheral blood mononuclear cells of elderly subjects. Thirty elderly subjects volunteered to participate in an 8-week resistance training program, and 11 youth subjects were included for basal assessments. Klotho, heat shock protein 60 (HSP60), oxidative marker expression (catalase, glutathione, lipid peroxidation, nuclear factor erythroid 2-related factor 2, protein carbonyls, reactive oxygen species, and superoxide dismutase 1 and 2), the IRE1 arm of UPR, and TLR4/TRAF6/pIRAK1 pathway activation were evaluated before and following training. No changes in the HSP60 and Klotho protein content, oxidative status markers, and TLR4/TRAF6/pIRAK1 pathway activation were found with exercise. However, an attenuation of the reduced pIRE1/IRE1 ratio was observed following training. Systems biology analysis showed that a low number of proteins (RPS27A, SYVN1, HSPA5, and XBP1) are associated with IRE1, where XBP1 and RPS27A are essential nodes according to the centrality analysis. Additionally, a gene ontology analysis confirms that endoplasmic reticulum stress is a key mechanism modulated by IRE1. These findings might partially support the modulatory effect of resistance training on the endoplasmic reticulum in the elderly.

## 1. Introduction

Aging and related diseases are associated with alterations in oxidative status [1] and low-grade inflammation [2], as well as a decreased endoplasmic reticulum (ER) unfolded protein response (UPR) [3,4]. Elevated oxidative stress may appear as a result of the imbalance between the antioxidant cell status and the physiological production of reactive oxygen species (ROS) [1]. Importantly, ROS have been shown to act as signaling molecules in DNA damage, and higher ROS levels might contribute to tumorigenesis, such as breast cancer [5,6,7]. Additionally, altered oxidative stress plays a key role in oxidative protein folding [8]. The H_2_O_2_/ROS-dependent reversible oxidation/reduction of the nucleophilic thiol side chain of specific cysteine residues is crucial in redox signaling [9]. This event causes structural changes and functional interactions through protein disulfide bond formation [9]. However, it could also lead to protein aggregation, resulting in ER stress and UPR activation [9]. Glutathione (GSH) is required to maintain ER oxidoreductases in a reduced state so that these enzymes can catalyze reduction or isomerization reactions, and its expression is upregulated by the NF-E2-related factor 2 (NRF2) transcription factor in response to UPR activation [10]. With age-related physiological changes, there is an inability to handle protein folding, accumulation, and aggregation due to a progressive failure of the chaperoning systems [11], as well as a decline in UPR components [12,13,14], ultimately leading to the perturbation of ER homeostasis. One main chaperone associated with the aging process is the mitochondrial heat shock protein 60 (HSP60) [15], which has been demonstrated to play a protective role in the maintenance of cellular homeostasis and protein refolding. However, the overexpression of HSP60 during aging could promote ER stress and the disruption of mitochondrial proteostasis [16,17], and also serve as an inflammatory mediator through the Toll-like receptor 4 (TLR4)-myeloid differentiation factor 88 (Myd88)-nuclear factor-kappa B (NF-kB) signaling pathway [18]. Specifically, upon TLR4 activation, a complex of tumor necrosis factor (TNF) receptor-associated factor 6 (TRAF6) and NADPH oxidase 2 (NOX2) is formed to promote the production of ROS, thereby eliciting the activation in inositol-requiring enzyme 1 (IRE1) of the UPR [19,20]. In this regard, an antiaging-associated protein, Klotho, has been proposed as an intermediate of these aging-related cellular processes via the downregulation of the TLR4/NF-κB pathway [21] and attenuation of ER stress [22] or oxidative stress [23].

Regular physical activity has been proposed as an effective intervention in the improvement of age-related diseases, such as osteoporosis [24], sarcopenia and dynapenia [25,26], cardiovascular diseases [27], type 2 diabetes [28], and reduced all-cause mortality [29]. In the elderly, resistance training has been demonstrated to attenuate the inflammatory response through the TLR2 and TLR4 pathways and Nod-like receptor pyrin domain-containing 3 (NLRP3) inflammasome activation [30,31]. Moreover, resistance training has been shown to ameliorate redox homeostasis by decreasing myeloperoxidase, thioredoxin reductase (TrxR)1, and manganese superoxide dismutase (MnSOD, also known as SOD2) protein expression [32]. This physiological adaptation to resistance training also improves the UPR by stimulating protein kinase R (PKR)-like ER kinase (PERK) and IRE1 pathways in elderly individuals [4]. However, the literature is limited with regard to investigating the effects of resistance training on the expression of HSP60 and Klotho in older adults. Therefore, the purpose of this study was to examine whether or not an 8-week resistance training program would modulate the oxidative status, the UPR activation, and the TLR4/TRAF6/pIRAK1 (phosphorylated interleukin-1 receptor-associated kinase 1) inflammatory pathway, along with their relationships with HSP60 and Klotho proteins in peripheral blood mononuclear cells (PBMCs) of elderly subjects. Furthermore, this study also utilized an in silico analysis to predict the key proteins associated with these biomolecules underlying physiological adaptations to exercise.

## 2. Materials and Methods

### 2.1. Subject Characteristics

Thirty healthy elderly (12 males (72.9 ± 0.52 years) and 18 females (73.2 ± 0.52 years)) and 11 young (males; 22.4 ± 2.2 years) subjects volunteered to participate in the study. The exclusion criteria included subjects with any known inflammatory diseases/conditions (e.g., dyslipidemia, hypertension, and diabetes mellitus) or the use of any medication known to affect the inflammatory status in the past six months before or during the study. Moreover, none of young female participants was included to avoid a potential hormonal effect (e.g., estrogen) on the measurement of outcome variables [33]. Participants were inexperienced with resistance training but maintained their regular exercise routines throughout the study. Before participating in this study, all subjects completed the informed consent form. A physician performed a medical anamnesis and general examination, including anthropometry, blood pressure measurements, and a baseline electrocardiogram, on each participant to rule out pathologies that might discourage participation in the study. Subsequently, elderly subjects were randomly assigned to either an 8-week resistance training group (TG; *n* = 22:11 males and 11 females) or a control group (CG; *n* = 8:1 male and 7 females). The principles of the Declaration of Helsinki were followed, and all procedures were approved by the local ethics committee (protocol code # 25-2013).

### 2.2. Maximal Strength Assessment

The BH^®^ Fitness Nevada Pro-T machine (Madrid, Spain) was used to perform the one-repetition maximum (1RM), setting the angle between the plane of the seat and the back to 100°. Elderly subjects started with a general warm-up of ten repetitions using 40% of their body weight. Once this weight was adequately lifted, the subject attempted two repetitions, increasing the weight from approximately 5 to 20 kg and continuing until a 1RM lift was determined. All increases in weight during the 1RM test were assessed using the rating of perceived exertion (RPE) OMNI-RES (0–10) scale [34], with a 3 min rest period between each attempt. Prior to performing the 1RM leg extension test with a 90° knee flexion, the lever arm of the knee extension machine was lined up with the center of rotation of the knee joint, and the tibial pad was adjusted for each subject proximal to the medial malleolus in the lower extremity. Starting at 90°, the knee joint’s range of motion ended at approximately 180°. Following a 10 min recovery, the elderly participants performed the 1RM seated bench press test with both arms in abduction at 90°, elbows bent at 90°, and the elbow joint’s range of motion ending at approximately 180°.

### 2.3. Resistance Exercise Training

The resistance training protocol consisted of 16 sessions over 8 weeks (2 sessions per week), with a minimum of 48 h between sessions. The participants started with a 10 min warm-up on a cycle ergometer. Subsequently, 8 different resistance exercises (leg press, ankle extension, bench press, leg extension, bicep curl, pec deck, high pulley traction, and dumbbell lateral lift) were performed using the exercise device. For each exercise, participants performed 3 sets of 12-8-12 repetitions. The first four exercises started with a 40% 1RM load, followed by an increase of 5% each week. For the last four exercises, the RPE with the OMNI-resistance exercise scale from 1 (light) to 10 (intense) was employed to prescribe the load as follows: an intensity of scale 5 during the first week, an intensity of scale 6 during the next three weeks, and an intensity of scale 8 for the last four weeks [35]. There was a 2–3 min rest between each repetition and a 3 min rest between each exercise.

### 2.4. Blood Sampling

Venous blood samples of the elderly were collected approximately 5–6 days before and after the training period and just before the training intervention in young subjects. The blood extraction was always carried out between 08:00 a.m. and 09:00 a.m. to avoid circadian effects following an overnight fast. An antecubital vein puncture was performed, and 30 mL of blood was collected using the ethylenediaminetetraacetic acid (EDTA) anticoagulant Vacutainer^TM^ systems (BD, Franklin Lakes, NJ, USA). Any strenuous exercise was requested to be avoided before blood draws, and no caffeine or alcohol was allowed during the intervention. From whole blood, the plasma and PBMC were obtained and stored at −80 °C for further analysis.

### 2.5. Isolation of PBMCs

The plasma fraction was separated from the cell phase by a 10 min centrifugation of the blood samples at 1500× *g* at 4 °C. Then, the cellular fraction was layered over a Ficoll separation solution (Biochrom AG, Berlin, Germany) and centrifuged at 240× *g* for 40 min at room temperature, and the PBMC interface was washed in phosphate-buffered saline, pH 7.4. PBMC lysis was performed in a pH 7.4 buffer, consisting of 10 mM Tris, 1 mM EDTA, and 0.25 mM sucrose, to which protease (cOmplete^TM^ Mini EDTA-free Protease Inhibitor Cocktail, Roche, Switzerland) and phosphatase (Sigma-Aldrich, St. Louis, MO, USA) inhibitor cocktails were added. The total protein concentration was obtained with the Bradford assay (cat# 5000006, Bio-Rad, Hercules, CA, USA).

### 2.6. Markers of Oxidative Status

The various oxidative stress biomarkers in PBMCs were analyzed using commercial kits, according to the manufacturers’ instructions. The protein carbonyl (PC, cat#: ab235631) fluorometric and lipid peroxidation (LP, cat#: ab233471) colorimetric assay kits were purchased from Abcam (Cambridge, UK). The reduced glutathione (GSH) colorimetric assay kit (cat# E-BC-K030-M) was obtained from Elabscience Biotechnology (Houston, TX, USA). The level of total reactive oxygen species (ROS) in PBMCs was measured using 10 µM of a 2-,7-dichlorodihydrofluorescein diacetate fluorescence probe (DCFH_2_-DA, cat# D6883, Millipore Sigma) as described previously [36]. An amount of 5 µL of the PBMC protein sample was analyzed for each biomarker, and the end-product was detected using a BioTek Epoch™ microplate spectrophotometer at 485 nm excitation and with 535 nm emission filters using a Synergy HTX spectrofluorometer (BioTek Instruments, Winooski, VT, USA). The data were normalized with the protein content of lysates.

### 2.7. Western Blot Analysis

Sodium dodecyl sulfate-polyacrylamide gel electrophoresis (SDS-PAGE) was used for fractionating samples containing 40 μg of protein on 4–20% Criterion™ TGX™ Precast gels (cat# 5671095, Bio-Rad). Once separated, the proteins were transferred to Immobilon-P polyvinylidene fluoride (PVDF) membranes (Millipore, Burlington, MA, USA) using the Trans-Blot Turbo Transfer System (Bio-Rad). Afterward, nonspecific binding was blocked by incubating membranes in 5% non-fat milk for 60 min at room temperature. Next, membranes were incubated with specific primary antibodies overnight at 4 °C [37]: catalase (Ref. sc-50508), HSP60 (Ref. sc-376240), NRF2 (Ref. sc-722), SOD1 (Ref. sc-101523), SOD2 (Ref. sc-18503), and TLR4 (Ref. sc-293072) (Santa Cruz Biotechnology, Dallas, TX, USA); IRE1 (Ref. ab37073), and pIRE1 (Ref. ab48187) (Abcam); TRAF6 (Ref. 8028) and β-tubulin (Ref. 3700) (Cell Signaling Technology, Danvers, MA, USA); and pIRAK1 (Ref. OAAF00238) (AVIVA Systems Biology, San Diego, CA, USA). Subsequently, they were incubated with the corresponding horseradish peroxidase-conjugated secondary antibodies for 1 h at room temperature: horse anti-mouse IgG (Ref. 7076, Cell Signaling Technology) and goat anti-rabbit IgG (Ref. 7074 Cell Signaling Technology). The reactive bands of the bound antibodies were detected in the ChemiDoc^TM^ XRS+ imaging system (Bio-Rad) by the addition of the SuperSignal™ West Pico Plus chemiluminescent substrate solution (cat# PI34580, Thermo Fisher, Waltham, MA, USA). An imaging densitometer (ImageJ, Bethesda, MD, USA) was used for the quantification density of the specific bands. Finally, densitometric analyses were normalized against the housekeeping protein β-actin.

### 2.8. Statistical Analysis

The Statistical Package for the Social Sciences (SPSS) version 26.0 (SPSS Inc., Chicago, IL, USA) was used to perform the statistical analyses. The normal data distribution was verified using the Shapiro–Wilk test. Independent *t*-tests were used to detect baseline differences between young vs. older adults and the CG vs. TG. In order to examine the effect of an 8-week resistance training on the values of catalase, HSP60, Klotho, NRF2, SOD1, SOD2, TLR4, TRAF6, pIRAK1, pIRE1/IRE1 ratio, LP, GSH, and PC in PBMCs, a repeated-measures analysis of variance (ANOVA) for group (trained vs. control) and time (pre vs. post) was utilized. If sphericity assumptions were violated, the Greenhouse–Geisser correction of degrees of freedom was used, and the Bonferroni method was used to compensate for multiple post hoc comparisons. Values are presented as mean ± standard error of the mean (SEM), with the statistical significance being *p* < 0.05.

### 2.9. In Silico Analysis

#### 2.9.1. Network Design

Input data to predict a protein–protein (PPI) network representative of the TLR4 pathway, IRE1 pathway, and antioxidant stress gene responses were based on gene ontologies (GOs) from the Molecular Signatures Database (MSigDB) [38]. The metasearch engine STRING 10.5 [39] was used for network design, considering the parameters: “text mining”, “experiments”, “databases”, and “coexpression”. The confidence value of interactions was 0.4. Nonconnected nodes were excluded from the network.

#### 2.9.2. Clustering and Gene Ontology Predictions

MCODE [40] and Cytoscape 3.7.2 [41] were used to predict densely connected regions (clusters) in the network. Afterward, the Cytoscape ClueGO 2.5.8 plugin [42] was applied for the enrichment network analysis of predicted clusters. This plugin is based on updated annotation files, including GO-predicted pathways. The calculation of this enrichment used the hypergeometric test, and a false discovery rate (FDR) adjusted *p*-value threshold of 0.0001.

#### 2.9.3. Centrality Parameter Analysis

Degree, betweenness, and eigenvector parameters were predicted from the PPI network using the Cytoscape platform, CentiScaPe 2.2 [43]. The centrality degree corresponds to the number of adjacent nodes connected to another unique node. In this work, the average of this parameter is the sum of different node degree scores divided by the total number of connections of the analyzed network. Another centrality parameter, betweenness, was explored. This parameter corresponds to the number of shortest paths between two nodes that pass through a targeted node. Similar to the degree average parameter, the betweenness average was calculated. Finally, the eigenvector was utilized to measure a given node’s regulatory potential based on its neighbors’ relevance. Nodes showing above-average scores for the node degree analysis are named Hub (H), nodes showing above-average scores for the betweenness analysis are denoted as Bottleneck (B), and those with above-average scores for the eigenvector analysis are termed Switch (S). Thus, the nodes of H, B, and S represent robust networking [43,44]. Venn diagrams were obtained using an online Venn tool (http://bioinformatics.psb.ugent.be/webtools/Venn/).

## 3. Results

### 3.1. Anthropometric and Strength Measurements of the Study Participants

The baseline anthropometric characteristics of the young and both elderly (CG and TG) groups are reported in Table 1.

At baseline, our analyses did not show any differences in both the 1RM bench-press seated (*p* = 0.929) and leg extension (*p* = 0.107) strength assessments between the CG and TG. However, following an 8-week resistance training protocol, a significant improvement in the 1RM bench-press seated and leg extension strength was observed (Table 2).

### 3.2. Oxidative Status, Inflammatory Response, and UPR Pathways at Baseline and Following 8-Week Resistance Training

**Figure 1:**
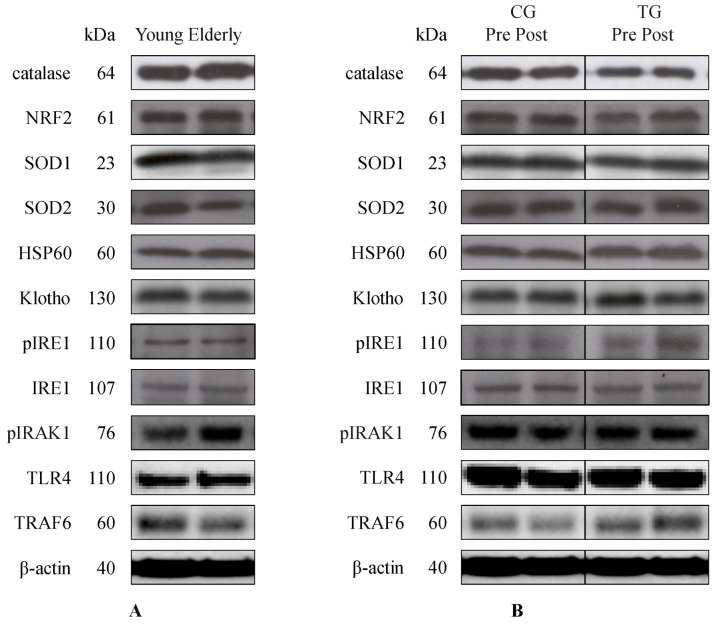
**Representative Western blots showing the effects of aging** (young, *n* = 11 (M); elderly, *n* = 30 (18/12 F/M)) (**A**) and resistance training (CG, *n* = 8 (7/1 F/M); TG, *n* = 22 (11/11 F/M)) (**B**) on catalase, NRF2, SOD1, SOD2, HSP60, Klotho, pIRE1, IRE1, pIRAK1, TLR4, and TRAF6 protein expression in PBMCs. CG, control group; F, female; M, male; TG, training group.

As shown in Table 3, no significant difference was found in the baseline levels of inflammatory proteins (pIRAK1, TLR4, and TRAF6) between young and elderly groups. Moreover, our analyses did not demonstrate any significant effects of aging on the expression of oxidative stress markers (catalase, GSH, NRF2, PC, SOD1, and ROS) at baseline. Additionally, the baseline values of protein folding-related HSP60 and the pIRE1/IRE1 ratio were comparable between both groups. Finally, our results did not show any significant effect of aging in the concentration of Klotho, whereas elderly subjects exhibited higher levels of LP along with a lower level of SOD2 in these oxidative status markers when compared to young subjects at baseline (Table 3/Figure 1A).

Additionally, both the CG and TG did not demonstrate any differences in the basal protein content of catalase, GSH, HSP60, Klotho, LP, pIRAK1, pIRE1/IRE1 ratio, ROS, SOD1, SOD2, and TLR4. However, a significant difference was found in the NRF2, PC, and TRAF6 protein expression between both groups (Table 4/Figure 1B).

Following an 8-week resistance training program, this study did not show any group × time effect in the protein expression of either inflammatory-related proteins (pIRAK1, TLR4, and TRAF6) or oxidative stress markers (catalase, GSH, LP, NRF2, PC, ROS, SOD1, and SOD2). It is important to note that the TG elicited attenuation of the reduced pIRE1/IRE1 ratio when compared to the CG following training (Table 4/Figure 1B). Additionally, our analyses did not demonstrate the training effect on the expression of HSP60 and Klotho or their relationships with other outcome variables. Finally, although elderly male and female subjects were included in the training program, no sex effect was found in this study.

### 3.3. Systems Biology Analysis of TLR4, IRE1, and Oxidative Stress Pathways

**Figure 2:**
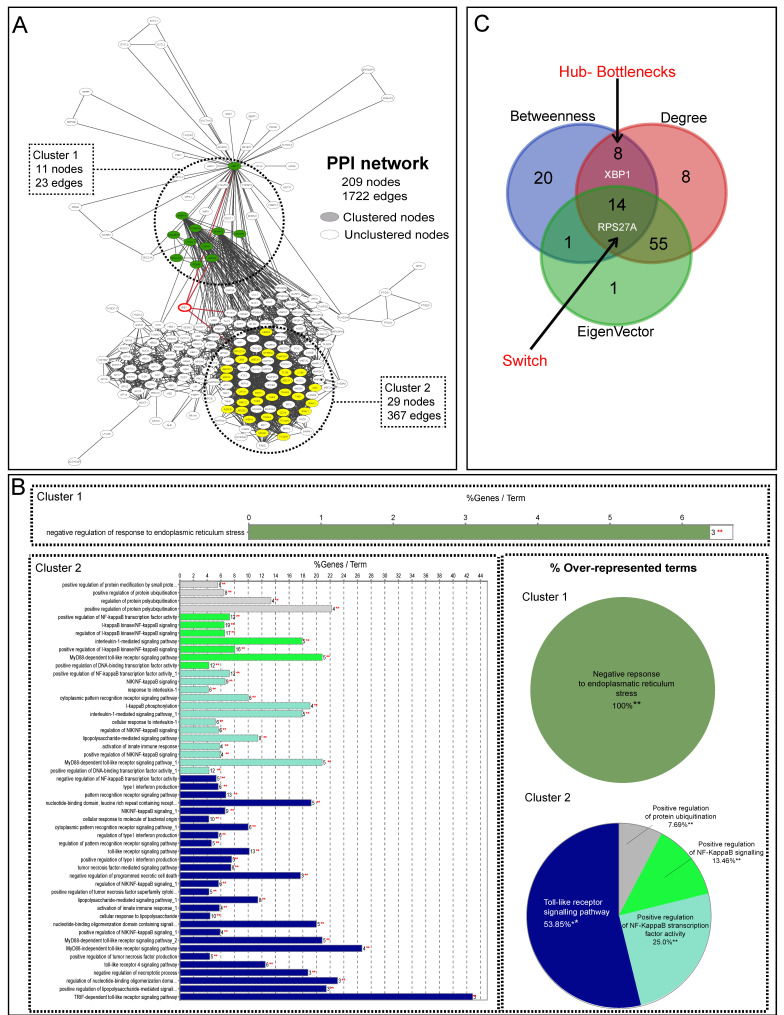
**Graph representing PPI network and topological analysis.** (**A**): Significant modules from the network are enclosed in dashed lines. These were obtained from MCODE analysis. (**B**): Subsequently, GO analysis was performed with ClueGO algorithms. ClueGO results with a pie chart of significant biological findings are represented. Functional enrichment for a given category was assessed by hypergeometric distribution. (**C**): Venn diagram analyzing the PPI network’s degree, betweenness, and eigenvector parameters.

Data on molecular entities involved in TLR4, IRE1, and oxidative stress pathways were extracted from the MSigDB, where 245 proteins were considered as the initial input to establish a PPI network. After STRING analysis, a PPI network composed of 209 nodes and 1722 edges was obtained (Figure 2A). In this network, the IRE1 node directly interacts with four proteins (ubiquitin-40S ribosomal protein S27a (RPS27A), E3 ubiquitin-protein ligase synoviolin (SYVN1), heat shock protein family A (HSP70) member 5 (HSPA5), and X-box-binding protein 1 (XBP1)). Afterward, the network’s global clustering organization was analyzed to determine if IRE1 is part of some dense region. The MCODE plugin was then applied, identifying two interconnected clusters, cluster 1 (11 nodes and 23 edges) and cluster 2 (29 nodes and 367 edges) (Figure 2B). Interestingly, IRE1 is an unclustered node, but it modulates two clustered proteins (RPS27A and XBP1). ClueGO was used to predict discrete biological processes associated with these clusters. In this regard, the term identified by the gene ontology analysis (GO) was a negative response to ER stress for cluster 1. On the other hand, four terms that were identified for cluster 2 were: (1) Toll-like receptor signaling pathway (53.85%); (2) positive regulation of NF-κB transcription factor activity (25%); (3) positive regulation of NF-kappa B signaling (13.46%), and (4) positive regulation for protein ubiquitination (7.69%).

Subsequently, the centrality analysis was performed to predict H-B-S nodes (Figure 2C and Supplementary Table S1). IRE1 is not an H, B, or S node, but the RPS27A node was identified as H-B-S, which is directly modulated by IRE1. In addition, XBP1 was also identified as H–B and associated with IRE1 (Figure 2C). Hub nodes with many interactions are mostly considered essential genes and make a network more vulnerable upon removal. Moreover, such nodes frequently occupy central positions in a network as they connect different network modules. On the other hand, Bottleneck and Switch nodes are points with higher flow information, and deleting them affects all network structures [45].

## 4. Discussion

The results demonstrated that the levels of the inflammatory proteins (pIRAK1, TLR4, and TRAF6), as well as different markers of the redox balance (catalase, GSH, LP, NRF2, PC, ROS, SOD1, and SOD2) remained unchanged with training. Although no changes in the protein content of HSP60 and Klotho proteins were observed, the TG exhibited a diminishment of the reduced pIRE1/IRE1 ratio when compared to the CG. These findings might partially support the beneficial effect of resistance training on the maintenance of cellular homeostasis and alleviation of ER stress in relatively healthy older adults.

Our findings agree with previous research [46,47], demonstrating a disturbance in the equilibrium of ROS production with a higher baseline level of LP in elderly vs. young adults. Although numerous studies have shown an age-dependent variation of GSH in many tissues, including lymphocytes (reviewed in Maher [48]), we did not find a significant difference in PBMCs between young and elderly subjects.

Other studies have reported unchanged values of GSH in the plasma from elderly humans [49,50] and in aged mouse liver [51], whereas GSH concentration was elevated in rat skeletal muscles [52]. The observation of these GSH results may depend on the analyses of respective tissues, as well as be a result of an attempt to restore GSH homeostasis [53]. Additionally, although this study found similar results in SOD1 expression, a reduction in SOD2 at baseline in elderly vs. young subjects was observed. A decrease in SOD1 activity has been shown in multiple tissues or cells, including human skin fibroblasts, skeletal muscle, lymphocytes, and erythrocytes in the elderly [54,55,56,57,58]. However, some studies did not demonstrate the effect of aging on the expression of the SOD1 protein [59,60,61,62,63], whereas SOD1 species were augmented in the spinal cord in aged mice [64] as well as in human plasma in elderly people [65]. In contrast, elderly subjects exhibited a lower level of SOD2 than young subjects in this study. Controversial results for SOD2 have also been found in the literature, showing an increase in aged human/rat skeletal muscles and rat liver [55,63,66,67], no changes in aged human leukocytes or rat kidneys [62,68], and a reduction in either skeletal muscles of aged mice [69] or the brains, hearts, livers, and kidneys of aged rats [70]. Taken together, these findings indicate that the expression of these antioxidant enzymes may be in a tissue-dependent manner.

Our previous studies have shown that an 8-week resistance training program is able to reduce NLRP3 inflammasome activation, induce the UPR, and increase mitochondrial biogenesis [4,31]. Here, we report that this resistance exercise program had no effect on the TLR4-TRAF6-pIRAK1 inflammatory pathway, supporting our previous results of no changes in the miR-146a [71]. Similarly, no changes in oxidative stress markers were found in this study. However, we corroborate the activation of the UPR, as evidenced by the attenuation of the reduced pIRE1/IRE1 ratio with training.

The interaction between oxidative stress and other stress signaling is complex. It has been suggested that altered ER redox homeostasis could induce ROS production in both ER and mitochondria [72]. Moreover, exercise-modulated ROS production could act as linking elements in the intracellular signaling mechanisms, triggering transcription factors as the peroxisome proliferator-activated receptor gamma coactivator 1-alpha (PGC1-α) [73]. In our previous work, we reported that the current resistance training protocol was able to increase PGC1-α expression, as well as mitofusin 1 [4]. The increase in mitochondrial biogenesis and fusion and decreased mitochondrial turnover could result in increased ROS production [74]. However, although we did not observe any change in total ROS levels, we cannot ensure that there are no changes in cellular organelles, such as mitochondria, sarcoplasmic reticulum, or peroxisomes, as the source of exercise-mediated ROS is not well-defined [73]. In addition, the crosswalk between cell signaling pathways modulated by exercise-mediated ROS and consequential physiological adaptations is not well-described. However, the improvement of antioxidant capacity with training could disrupt ROS-mediated signaling [75]. Overall, resistance training in the elderly has been reported with different outcomes depending on the health status of the individuals, tissue sources, and the training duration or intensity [76,77]. It is important to note that the use of biochemical analyses could be a critical point in the evaluation of in vivo oxidative stress, due to its potential unreliability and inaccuracy [78]. Thus, the reactive oxygen metabolite derivative compounds (d-ROMs) and biological antioxidant potential (BAP) tests that utilize the capillary blood samples from the fingertip can provide a measure of the whole oxidant capacity and the biological antioxidant potential in plasma [79,80].

The present research found no changes in either the Klotho or HSP60 protein in PBMCs with resistance training. It has been proposed that the expression of oxidative stress-mediated ROS could be modulated by the antiaging gene Klotho through FoxO3a (forkhead box protein O3) phosphorylation [81], and this modulation could be driven through NFR2 [82,83] as well as via NF-κB activation [84,85]. While this study did not observe significant differences in either NRF2 or TRAF6-pIRAK1 between young and elderly subjects, several studies have reported an increase in the soluble form of plasma Klotho (s-klotho) in sedentary middle-aged adults [86] and postmenopausal women [87] following 12 weeks of aerobic training. In this regard, aerobic training has also been supported to enhance Klotho expression and attenuate ROS production in rat brain and kidney [88]. Since research has recently proven that Klotho overexpression prevents ER stress-induced IRE1 response in senescent monocytes [89] and human renal epithelial HK-2, alveolar epithelial A549, HEK293, and SH-SH-SY5Y neuroblastoma cells [22], it would be reasonable to hypothesize that the current resistance training protocol did not promote an increase in Klotho expression to avoid a lower IRE1 response in the elderly. Although HSP60 is known to contribute to the refolding of mitochondrial proteins, its role in aging and related diseases still needs to be fully elucidated [15]. Increased protein levels of basal HSP60 have been shown in skeletal muscle in aerobically trained individuals [90] and following acute aerobic protocols [91,92]; however, no changes in HPS60 protein content were reported in both the muscle and plasma of aged mice after a 5-week endurance training program [93]. Further studies are needed to investigate the potential regulatory roles of Klotho and HSP60 on inflammation, ER stress, and changes in oxidative balance in response to exercise in the elderly.

Although the beneficial effects of regular physical exercise to alleviate inflammation and oxidative stress is well-established [94,95], the processes of these physiological adaptations with regard to the UPR remains to be explored. Thus, a systems biology approach was utilized to define putative targets of IRE1. The network analysis showed four interactors of IRE1: XBP1, HSPA5, SYVN1, and RPS27A in the initial two clusters. The first cluster is directly related to ER stress through XBP1, which we had previously found to be increased in PBMCs of elderly subjects in response to an 8-week resistance training program [4]. Upon activation, pIRE1 mediates XBP1 mRNA splicing (XBP1s) through its kinase activity [96], providing a cytoprotective role in contrast to IRE1 endoribonuclease activity-mediated RIDD (regulated IRE1-dependent decay) that takes part in cell fate regulation [97]. Translated XBP1s transactivates gene expression related to protein folding, ER quality control, and ER-associated protein degradation (ERAD) components [98], and regulates the transcription of genes involved in lipid and glucose metabolism [99] and immune responses [100]. However, although XBP1s is located in the nucleus, unspliced XBP1 (XBP1u) is found in the cytoplasm to not only involve in ER stress, but also help maintain other physiological functions [101], including: (1) the regulation of autophagy through FoxO1 interaction [102]; (2) cellular redox homeostasis via Nrf2-promoted transcription of heme oxygenase 1 (HO-1) in response to serine/threonine-protein kinase mTOR-mediated RAC-alpha serine/threonine-protein kinase (Akt1) phosphorylation [103]; (3) the promotion of tumorigenesis, leading to p53 ubiquitination/proteasomal degradation [104]; and (4) the regulation of vascular smooth muscle cells’ phenotypic transition, maintenance of vascular homeostasis [105], and the inhibition of vascular calcification [106].

On the other hand, IRE1 interacts with or controls several essential proteins, such as RPS27A, that act as mediators of cellular communication. Specifically, RPS27A is a core ribosomal protein related to the second IRE1-modulated cluster. This is a novel mechanism to be explored since there is no scientific research evaluating how IRE1 interacts with RPS27A, which is involved in ribosome composition, 40S ribosomal maturation, or the cell cycle through p53/p21 modulation [107]. Interestingly, ubiquitin is also encoded by the RPS27A gene [108] and plays a major role in protein homeostasis, specifically targeting unneeded cellular proteins for proteasomal degradation [109]. Although it is known that ERAD mediates the degradation of misfolded proteins in the ubiquitin–proteasome system [110], the modulatory role of IRE1 remains completely unknown.

Remarkably, the IRE1/XBP1 arm of the UPR has been shown to act independently of the other branches and plays a central role in the regulation of macrophage activation [111]. The TLR-mediated IRE1/XBP1 induction occurs in the absence of an ER stress response, whereas the TLRs and IRE1 engagement-induced XBP1 activation promotes sustained production of inflammatory mediators, such as interleukin (IL)-6 and TNF-α [111]. Moreover, a feedback loop of TNF-α-induced XBP1 in rheumatoid arthritis synoviocytes has been observed [112]. Nonetheless, ER stress-induced IRE1 has also been reported to modulate cytokine production differently, including in the production of IL-1β and TNF-α via the activation of glycogen synthase kinase (GSK)-3β and XBP1, respectively [113]. Finally, IRE1 has been proven to be a key interactor involved in NF-κB activation [114,115] and might have a fundamental role in metabolic syndrome and lipid disorders [116], regenerative myogenesis [117], or cardiac diseases [118]. The upregulation of this IRE1-mediated NF-κB has also been shown to enhance NLRP3 expression in a liver fibrosis model [119].

## 5. Conclusions

This study demonstrated an attenuation of the reduced pIRE1/IRE1 ratio in exercised elderly subjects, supporting the effect of an 8-week resistance training program on the maintenance of the IRE1 arm of the UPR. Although only limited significant changes were reported in this study, the approach of our in silico analysis further supports physiological adaptations to exercise in older adults. However, the measurement of these variables in skeletal muscle is required to better understand how exercise could alleviate the perturbation of ER homeostasis that results in the accumulation of unfolded proteins, and potentially, redox imbalance in the elderly.

## Figures and Tables

**Table 1:** Participant anthropometric characteristics.

	Young (N = 11)	CG (N = 8)	TG (N = 22)	
	Mean ± SEM	Mean ± SEM	Mean ± SEM	*p*-Value
**Age (years)**	22.4 ± 2.2	74.13 ± 0.9	72.7 ± 0.4	0.082
**Height (cm)**	176.3 ± 1.5	153.4 ± 2.8	164.9 ± 2.4	0.018 *
**Weight (kg)**	77.6 ± 1.8	69.6 ± 5.4	74.1 ± 3.6	0.540
**BMI (kg/m^2^)**	25.1 ± 1.4	29.6 ± 2.2	26.9 ± 0.9	0.192

CG = control group; TG = training group; the * indicates a significant difference at baseline between CG and TG.

**Table 2:** Effect of an 8-week resistance training in muscular strength.

	CG (Pre)	CG (Post)	TG (Pre)	TG (Post)	
	Mean ± SEM	Mean ± SEM	Mean ± SEM	Mean ± SEM	*p*-Value
**1RM bench-press seated (kg)**	55.1 ± 5.2	44.8 ± 3.1	54.5 ± 4.6	72.2 ± 5.0	<0.001 *
**1RM leg extension (kg)**	55.9 ± 6. 7	57.7 ± 4.6	72.4 ± 5.4	88.0 ± 5.6	0.042 *

CG = control group; TG = training group; 1RM = one-repetition maximum. The * indicates a significant difference following training between CG and TG.

**Table 3:** Oxidative status, UPR IRE1 pathway activation, and inflammatory response at baseline in young and elderly subjects.

	Young (N = 11)	Elderly (N = 30)	
	Mean ± SEM	Mean ± SEM	*p*-Value
**GSH (nM/mg protein)**	1.25 ± 0.02	1.33 ± 0.03	0.057
**LP (nM/mg protein)**	0.52 ± 0.02	0.66 ± 0.03	0.001 *
**PC (nM/mg protein)**	4.00 ± 0.37	5.05 ± 0.47	0.210
**ROS (DCF RFUX10^3^/mg protein)**	712.1 ± 53.7	716.1 ± 55.0	0.968
**Catalase (O.D.)**	0.90 ± 0.06	0.84 ± 0.04	0.476
**NRF2 (O.D.)**	0.79 ± 0.14	0.64 ± 0.05	0.311
**SOD1 (O.D.)**	1.17 ± 0.08	1.20 ± 0.10	0.871
**SOD2 (O.D.)**	0.93 ± 0.14	0.63 ± 0.07	0.037 *
**HSP60 (O.D.)**	0.82 ± 0.04	0.86 ± 0.03	0.511
**Klotho (O.D.)**	0.61 ± 0.11	0.86 ± 0.12	0.222
**pIRE1/IRE1 ratio (O.D.)**	1.45 ± 0.21	1.67 ± 0.12	0.347
**pIRAK1 (O.D.)**	0.46 ± 0.09	0.64 ± 0.06	0.115
**TLR4 (O.D.)**	0.58 ± 0.11	0.69 ± 0.69	0.361
**TRAF6 (O.D.)**	0.58 ± 0.09	0.43 ± 0.06	0.218

O.D. = optical density; the * indicates a significant difference at baseline between both groups.

**Table 4:** Oxidative status, UPR IRE1 pathway activation, and inflammatory response in the elderly at baseline and after an 8-week resistance training.

	CG (Pre)	CG (Post)	TG (Pre)	TG (Post)	*p*-Value	
	Mean ± SEM	Mean ± SEM	Mean ± SEM	Mean ± SEM	Baseline	Group × Time
**GSH (nM/mg protein)**	1.25 ± 0.06	1.33 ± 0.04	1.36 ± 0.03	1.40 ± 0.03	0.050	0.532
**LP (nM/mg protein)**	0.56 ± 0.06	0.65 ± 0.06	0.69 ± 0.04	0.65 ± 0.03	0.083	0.174
**PC (nM/mg protein)**	3.45 ± 0.67	4.00 ± 0.73	5.63 ± 0.56	5.05 ± 0.54	0.039 *	0.401
**ROS (DCF RFUX10^3^/mg protein)**	812.4 ± 148.2	1028.2 ± 231.1	681.1 ± 53.0	726.4 ± 47.8	0.299	0.419
**Catalase (O.D.)**	0.92 ± 0.09	1.08 ± 0.11	0.81 ± 0.05	0.81 ± 0.05	0.288	0.074
**NRF2 (O.D.)**	0.80 ± 0.10	0.82 ± 0.12	0.58 ± 0.05	0.49 ± 0.06	0.034 *	0.279
**SOD1 (O.D.)**	1.43 ± 0.24	1.19 ± 0.18	1.12 ± 0.10	1.24 ± 0.23	0.186	0.295
**SOD2 (O.D.)**	0.73 ± 0.17	0.79 ± 0.18	0.60 ± 0.07	0.61 ± 0.06	0.382	0.496
**HSP60 (O.D.)**	0.80 ± 0.06	0.88 ± 0.06	0.87 ± 0.04	0.93 ± 0.05	0.325	0.816
**Klotho (O.D.)**	1.02 ± 0.22	1.01 ± 0.16	0.81 ± 0.14	0.78 ± 0.16	0.437	0.930
**pIRE1/IRE1 ratio (O.D.)**	1.88 ± 0.25	1.53 ± 0.26	1.60 ± 0.13	1.69 ± 0.14	0.308	0.040 *
**pIRAK1 (O.D.)**	0.68 ± 0.14	0.59 ± 0.11	0.63 ± 0.07	0.56 ± 0.07	0.718	0.836
**TLR4 (O.D.)**	0.74 ± 0.12	0.78 ± 0.13	0.68 ± 0.06	0.68 ± 0.05	0.591	0.744
**TRAF6 (O.D.)**	0.64 ± 0.12	0.46 ± 0.09	0.36 ± 0.06	0.34 ± 0.06	0.037 *	0.167

CG = control group; O.D. = optical density; TG = training group. The * indicates a significant difference either at baseline or following training between CG and TG.

## Data Availability

Data is contained within the article and supplementary material.

## Supplementary Materials

The following supporting information can be downloaded at: https://www.mdpi.com/article/10.3390/antiox11112242/s1, Table S1: CentiScape Analysis.
